# The complete chloroplast genome sequence of *Prunus Cerasifera* Ehrh. ‘Pissardii’ (Rosaceae)

**DOI:** 10.1080/23802359.2019.1681314

**Published:** 2019-10-24

**Authors:** Yan Huo, Ming Yan, Xueqing Zhao, Zunling Zhu, Zhaohe Yuan

**Affiliations:** a College of Landscape Architecture, Co-Innovation Center for Sustainable Forestry in Southern China, Nanjing Forestry University, Nanjing, China;; b College of Forestry, Nanjing Forestry University, Nanjing, China;; c College of Arts and Design, Nanjing Forestry University, Nanjing, China

**Keywords:** *Prunus cerasifera*, *Prunus cerasifera* ‘Pissardii’, complete chloroplast genome, phylogenetic analysis

## Abstract

*Prunus cerasifer*a Ehrh. ‘Pissardii’, is a widespread ornamental and fruit tree. Here, we reported the complete chloroplast (cp) genome of *P. cerasifer*a ‘Pissardii’ (GenBank accession number: MN418903). The total cp genome is 157,952 bp in length, displayed a typical quadripartite structure, including a large single copy region (LSC) of 86,286 bp and a small single copy region (SSC) of 18,926 bp, which are separated by a pair of inverted repeat (IR) regions of 26,370 bp. The overall guanine-cytosine (GC) content of the genome sequence is 36.7%. The cp genome encodes 134 unique genes, including 84 protein-coding genes, 42 tRNA genes, and 8 rRNA genes. Phylogenetic analysis of 27 chloroplast genomes showed that *P. cerasifera* ‘Pissardii’ was closely related to *P. humilis* in Rosaceae.

*Prunus cerasifer*a Ehrh. (Rosaceae), commonly called Cherry Plum, is native to Southeast Europe and Western Asia. *P. cerasifer*a Ehrh. ‘Pissardii’ is a popular ornamental cultivar due to its unusual purple leaves, white to light pink springtime flowers and edible purple fruits (Zhang 2010). So far, there have been no studies on the complete chloroplast genome of *P. cerasifera* or *P. cerasifera* ‘Pissardii’. Considering that universal plastid markers cannot provide enough information to construct the phylogeny of *prunus* species (Horvath et al. 2011) and cp genome-scale data have proven to be useful in resolving phylogenetic relationships (Olmstead and Palmer 1994), we first report the complete chloroplast genome of *P. cerasifera* ‘Pissardii’, which will provide valuable molecular data for species identification and phylogenetic analysis for genus *Prunus*.

The total genomic DNA was extracted by the modified CTAB method (Doyle 1987) from fresh leaves that were collected from a single individual of *P. cerasifera* ‘Pissardii’ in Nanjing (latitude: 32°07′86.1″N, longitude: 118°81′69.4″E), Jiangsu Province, China. The voucher specimen deposited in Nanjing Forestry University (accession number NFU19051102). Paired-end libraries were constructed and sequenced with an Illumina Hiseq 2500 platform (Nanjing, China) for paired-end 150 bp reads. The cp genome was assembled via NOVOPlasty (Dierckxsens et al. 2017), using the *p. cerasoides* cp genome (GenBank accession MF621234) as a reference. The finished cp genome was annotated by GeSeq (Tillich et al. 2017), coupled with manual adjustment. Geneious 8.0.4 (Kearse et al. 2012) was used for inspecting the cp genome structure.

The complete cp genome size of *P. cerasifera* ‘Pissardii’ is 157,952 bp in length, including an LSC region of 86,286 bp and an SSC region of 18,926 bp, which are separated by a pair of IR regions of 26,370 bp. The overall GC content of the cp genome is 36.7% and those in the LSC, SSC, and IR regions are 34.5, 30.5, and 42.6%, respectively. The cp genome encodes 134 unique genes, including 84 protein-coding genes (PCGs), 42 tRNA genes, and 8 rRNA genes. The tRNA genes are distributed throughout the whole genome with 25 in the LSC, 1 in the SSC, 15 in the IR regions, while rRNAs only situate in IR regions. One gene (*trnM-CAU*) has three copies. Twenty-one genes have two copies, which include eight PCGs (*ndhB*, *rpl2*, *rpl23*, *rps12, rps19*, *rps7*, *ycf1*, *ycf2*), nine tRNA genes (*trnA-UGC, trnG-UCC, trnI-CAU, trnI-GAU, trnL-CAA, trnN-GUU, trnR-ACG, trnT-GGU, trnV-GAC*), and four all rRNA genes *(rrn16*, *rrn23*, *rrn4.5*, and *rrn5*). Among the PCGs, two genes (*ycf3* and *rps12*) contain two introns and 13 different genes (*atpF, clpP, ndhA, ndhB, rpl2, rpoC1, rps16, trnA-UGC, trnG-UCC, trnI-GAU, trnK-UUU, trnL-UAA, trnV-UAC*) have one intron each.

In order to investigate the evolutionary relationship, the phylogenetic tree including *P. cerasifera* ‘Pissardii’, 24 Rosaceae species and 2 outgroup species was constructed by cp genomes. Chloroplast genomes sequences were aligned with MAFFT (Katoh and Standley 2013). The maximum likelihood (ML) tree was performed using IQ-TREE (Nguyen et al. 2015). The result revealed that *P. cerasifera* ‘Pissardii’ was closely related to *P. humilis* in Rosaceae (Figure 1).

**Figure 1.**
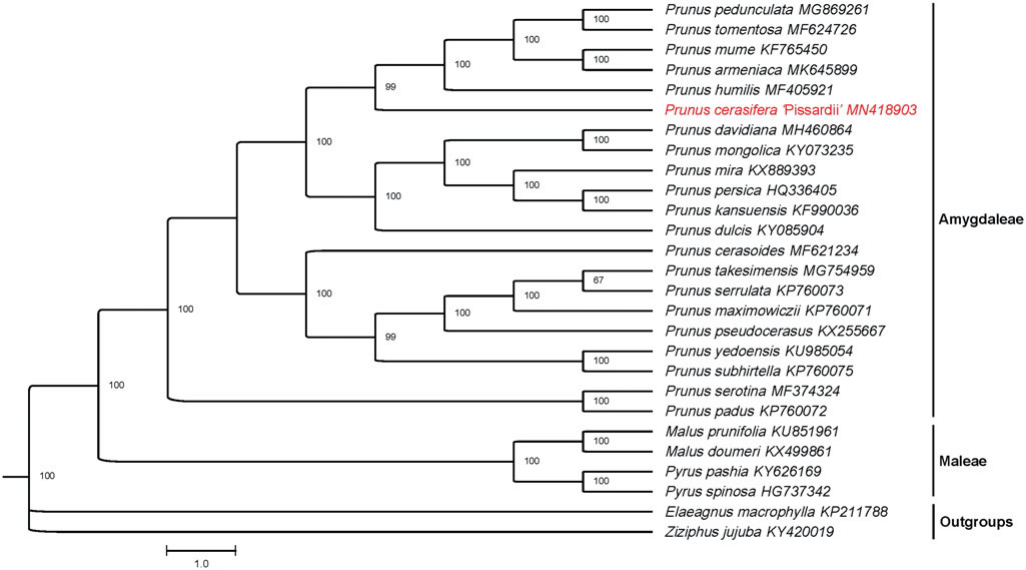
Maximum likelihood (ML) tree based on the chloroplast genome sequences of 27 species. *Elaeagnus macrophylla* and *Ziziphus jujuba* were selected as outgroups. Numbers on the nodes are bootstrap values from 1000 replicates.
